# Structure-guided discovery of novel AflG inhibitors for aflatoxin contamination control in *aspergillus flavus*

**DOI:** 10.3389/fmicb.2024.1425790

**Published:** 2024-07-12

**Authors:** Fenghua Wang, Weijie Zhou, Maohua Yang, Jinlu Niu, Wenjie Huang, Zhaofu Chen, Yuanyuan Chen, Dongdong Wang, Jun Zhang, Shaowen Wu, Shijuan Yan

**Affiliations:** ^1^College of Resources and Environmental Sciences, Gansu Agricultural University, Lanzhou, China; ^2^State Key Laboratory of Swine and Poultry Breeding Industry, Guangdong Key Laboratory for Crop Germplasm Resources Preservation and Utilization, Agro-biological Gene Research Center, Guangdong Academy of Agricultural Sciences, Guangzhou, China; ^3^DP Technology, Beijing, China

**Keywords:** aflatoxins, *aspergillus flavus*, P450 monooxygenase AflG, structure-guided inhibitor design, contamination control

## Abstract

Aflatoxins (AFs) are highly carcinogenic metabolites produced by *Aspergillus* species that can contaminate critical food staples, leading to significant health and economic risks. The cytochrome P450 monooxygenase AflG catalyzes an early step in AF biosynthesis, resulting in the conversion of averantin (AVN) to 5′-hydroxy-averantin. However, the molecular mechanism underlying the AflG-AVN interaction remains unclear. Here, we sought to understand the structural features of AflG in complex with AVN to enable the identification of inhibitors targeting the AflG binding pocket. To achieve this goal, we employed a comprehensive approach combining computational and experimental methods. Structural modeling and microsecond-scale molecular dynamics (MD) simulations yielded new insights into AflG architecture and unveiled unique ligand binding conformations of the AflG-AVN complex. High-throughput virtual screening of more than 1.3 million compounds pinpointed specific subsets with favorable predicted docking scores. The resulting compounds were ranked based on binding free energy calculations and evaluated with MD simulations and *in vitro* experiments with *Aspergillus flavus*. Our results revealed two compounds significantly inhibited AF biosynthesis. Comprehensive structural analysis elucidated the binding sites of competitive inhibitors and demonstrated their regulation of AflG dynamics. This structure-guided pipeline successfully enabled the identification of novel AflG inhibitors and provided novel molecular insights that will guide future efforts to develop effective therapeutics that prevent AF contamination.

## 1 Introduction

Aflatoxins (AFs) are highly toxic, carcinogenic secondary metabolites primarily produced by the fungi *Aspergillus flavus* and *Aspergillus parasiticus* (Caceres et al., 2020). These fungi are ubiquitous in the environment, leading to widespread aflatoxin contamination in agricultural products (Liao et al., 2020). When temperature and humidity conditions are favorable, AFs are produced in large volumes, contaminating supplies of staple foods such as maize, peanuts, and other nuts. This can occur at multiple points in production, including the growth, harvest, storage, and processing stages (Jallow et al., 2021). Consumption of AF-contaminated foods by humans or other animals can result in various disorders and diseases, such as nutritional malabsorption, infertility, endocrine disruption, congenital fetal malformations, and immune deficiencies (Razzaghi-Abyaneh et al., 2014; Caceres et al., 2020). Mitigating AF contamination in crops is crucial to alleviate the associated health risks and economic impacts.

AF biosynthesis is tightly regulated by various factors, including nutrient sources, oxidative stress responses, ambient temperature, pH, and light conditions. Favorable conditions induce cellular signaling pathways that up-regulate genes involved in AF production (Bhatnagar et al., 2003; Georgianna and Payne, 2009; Caceres et al., 2020; Wu et al., 2022, 2023). The AF biosynthetic pathway contains 30 genes encoding both enzymes and regulatory factors. The enzymes in this pathway catalyze at least 27 reactions, including numerous oxidative rearrangements, making AF biosynthesis one of the most extensive and intricate biological processes known (Chang, 2003; Price et al., 2006; Caceres et al., 2020; Wang et al., 2022). Over a decade ago, researchers embarked on efforts to understand the functional mechanisms of enzymes in the AF biosynthetic pathway. For example, Crawford et al. solved the structure of the product template (PT) domain within PksA, a multidomain iterative polyketide synthase that initiates AF biosynthesis in *A. parasiticus*, and revealed the PT domain’s capacity to bind both linear and bicyclic polyketides (Crawford et al., 2009). Subsequently, the thioesterase/Claisen cyclase domain in PksA was found to have an α/β-hydrolase fold in the catalytic closed form, with a distinct hydrophobic substrate-binding chamber (Korman et al., 2010). Despite such mechanistic insights, the functional mechanisms of most enzymes in the AF biosynthetic pathway have remained elusive.

Previous studies in other biochemical pathways of interest have demonstrated the capacities of targeted inhibitors to regulate metabolic processes. For instance, asparaginase and glutaminase inhibitors have been utilized to modify amino acid metabolism in hematologic malignancies, effectively reversing immune suppression (Tabe et al., 2019). Inhibitors of monoamine oxidases (MAOs), which catalyze oxidative deamination of several neurotransmitters, modulate neurotransmitter metabolism and may act as therapeutics in those with Alzheimer’s disease by reducing harmful by-products and the associated oxidative stress (Manzoor and Hoda, 2020). Furthermore, several categories of compounds (namely amidepsines, roselipins, and xanthohumol) have been shown to inhibit the activity of diacylglycerol acyltransferase produced by the marine fungus *Gliocladium roseum*, regulating triacylglycerol biosynthesis (Sharma et al., 2020). These studies collectively indicate that inhibitors targeting key enzymes in specific metabolic pathways can effectively modulate the production of these metabolites, suggesting that AF production could be curtailed via targeted inhibition of critical enzymes in the AF biosynthetic pathway.

Building upon this concept, recent research has employed *in silico* docking methodologies to identify potential inhibitors of the AF biosynthetic enzyme PksA. In one such study, researchers conducted a virtual screening of 623 natural compounds from the South African natural compound database, identifying 10 molecules with predicted favorable binding energies (Labib et al., 2022). However, the multiple subunits of PksA, which catalyze various reactions, pose a challenge to effective PksA inhibition (Crawford et al., 2009). Thus, it may prove more fruitful to target other enzymes in the AF biosynthetic pathway. Earlier investigations have underscored the central roles of cytochrome P450s, a Heme-type superfamily of enzymes with a highly conserved basic fold, in AF biosynthesis (Uka et al., 2020). P450s have been rigorously investigated in prior studies as prospective targets for inhibitor design, laying the groundwork for future efforts (Sezutsu et al., 2013; Hussain et al., 2020; Stout et al., 2021). The P450 AflG is involved in an early step of AF biosynthesis, in which it catalyzes the conversion of averantin (AVN) to 5′-hydroxy-averantin (Yu et al., 1997; Uka et al., 2020). Thus, AflG may be a viable target for inhibitor design to prevent AF biosynthesis.

In the present study, we investigated the AflG-AVN interaction and identified potential AflG inhibitors using structural modeling, molecular dynamics simulations, virtual screening, binding free energy calculations, and experimental validation. This approach was designed to yield novel insights into the structural architecture of the substrate and enzyme and to identify crucial residues that govern their interaction. High-throughput docking was then used to screen a vast library of millions of compounds, allowing the identification of molecules with favorable docking scores. Subsequent binding free energy calculations were conducted to narrow the selection to a handful of candidate compounds that could be tested *in vitro*. This study establishes an effective framework for further development and optimization of effective AflG inhibitors, promoting the establishment of effective agricultural therapeutics to mitigate AF contamination.

## 2 Materials and methods

### 2.1 Structural modeling and molecular docking

The *A. flavus* strain NRRL 3357, obtained from the United States Department of Agriculture Agricultural Research Service (USDA-ARS) culture collection (Peoria, IL, United States), was selected for this study due to its extensively characterized genome and widespread use as a model organism in investigations of secondary metabolite regulation and biosynthesis (Nierman et al., 2015; Skerker et al., 2021). The AflG amino acid sequence from *A. flavus* strain NRRL 3357 and the corresponding structural model (predicted with Alphafold2) were obtained from UniProt (ID: B8NHZ0) (The UniProt Consortium, 2023). The validity of the model was evaluated using predicted local-distance difference test (pLDDT) scores and visual inspection. The initial 35 amino acids, predicted to interact with membranes, were excised from the AlphaFold2 structural model (Kufareva et al., 2014). The revised structure was then employed for subsequent construction steps. The structure of the closely related human enzyme P450 3A5 (PDB ID: 7sv2) was predicted with SWISS-MODEL and aligned to the AflG model using PyMOL (v2.5 Educational Edition, Schrödinger, LLC) (Waterhouse et al., 2018). The Heme cofactor from the aligned P450 3A5 structure was incorporated into the AflG model, forming the AflG-Heme complex.

CavityPlus was employed to detect potential binding pockets in the AflG-Heme complex, into which the native substrate AVN was docked (Wang et al., 2023). Polar hydrogens and Gasteiger charges were added to the receptor structure using the AutodockTools v.4.2 package (Morris et al., 2009). The AVN structure was obtained from PubChem and processed using the Python script “mk_prepare_ligand.py” from Meeko (Holcomb et al., 2022). The grid spaces around potential AflG binding pockets were defined in AutodockTools. The number of points was set to 50 Å in x-, y-, and z-dimensions. The center coordinates were − 10.35 Å, 36.078 Å, and 40.881 Å on the x-, y-, and z-axes, respectively. The grid spacing was 0.375 Å. The AutoDock force field affinity maps were generated using AutoGrid4 in AutodockTools. Molecular docking was conducted using Autodock-Vina and the AutoDock4 forcefield with default parameters (Eberhardt et al., 2021). A high-scoring docking pose, in which the AVN alkyl chain was positioned proximate to the Heme iron center, was selected as the AflG-Heme-AVN complex model.

### 2.2 MD simulations for AflG and the AflG-Heme-AVN complex

Two simulation systems were established to analyze AflG dynamics and interactions with AVN: AflG alone and the AflG-Heme-AVN complex. The initial structures for AflG and AflG-Heme-AVN were obtained as described in section 2.1. Water, Na^+^, and Cl^−^ were added to solvate and neutralize the systems at physiological salinity (150 mM). MD simulations were conducted in GROMACS 2020 using the all-atom CHARMM36m force field and the TIP3P water model (Abraham et al., 2015; Huang et al., 2017). Topology and parameter files for AVN were generated with a CHARMM generalized force field (CGenFF) (Vanommeslaeghe et al., 2010). The parameter file for Heme, specifying an Fe(II) oxidation state advantageous for oxygen binding, was sourced from CHARMM-GUI (Jo et al., 2008). The steepest descent algorithm was used for energy minimization over 50,000 steps. The system equilibration lasted 100 ps with constraints on the hydrogen bonds in the isothermal-isobaric (NPT) ensemble (Deift and Zhou, 1993). The semi-isotropic Parrinello-Rahman method was used to maintain the pressure at 1 bar with a time constant of 2 ps. The v-rescale method was used with a time constant of 0.1 ps to maintain a constant temperature of 298 K (Parrinello and Rahman, 1980). Trajectories were produced with constraints on the H-bonds and lasted 1 μs for both AflG and AflG-Heme-AVN complex. H-bonds were constrained using the LINCS algorithm at 2 fs intervals (Hess et al., 1997). The threshold values for electrostatic and van der Waals interactions were both 1.2 nm. Long-range electrostatic interactions were computed using the particle mesh Ewald (PME) method (Essmann et al., 1995). The root mean square deviation (RMSD), root mean square fluctuation (RMSF), and interaction fingerprint values of the MD trajectories were calculated with GROMACS modules and MD-IFP (Kokh et al., 2020). The Gromos algorithm was employed for cluster analysis, using a backbone RMSD threshold of 0.3 nm for AflG and 0.2 nm for the AflG-Heme-AVN complex.

### 2.3 Large-scale virtual screening

AVN was removed from the central structure of the top cluster in the AflG-Heme-AVN ensemble, and the resulting structure was used as the receptor in virtual screening. The screening compound library comprised 1.3 million molecules from the ZINC20 database with Log(*p*) values ≤3.5 and 32,000 molecules from TopScience Co. Ltd. (Shanghai, China). The docking-ready pdbqt files for each compound were downloaded from the ZINC20 database. The two-dimensional structures of molecules obtained from TopScience were converted to three-dimensional structures using OpenBabel v.3.1.0 and then processed with the Python script “mk_prepare_ligand.py” (O'Boyle et al., 2011). The receptor structure was prepared, the grid space was set, and the affinity maps were generated as described in section 2.1 with several exceptions: the point settings of the x-, y-, and z-dimensions were 52 Å, 32 Å, and 22 Å with centers at 4.057 Å, 2.767 Å, and 7.66 Å, respectively. The structure derived from the MD simulation exhibited a systematic deviation in position compared to the docked AflG-Heme-AVN model, rendering the grid box center positions incomparable. Autodock-GPU was used to dock molecules from the compound library using the default parameters (Santos-Martins et al., 2021). Molecules that exhibited a docking energy < −7 kcal/mol were classified as highly ranked compounds for further analysis.

### 2.4 Molecular mechanics/generalized born surface area calculations

Binding free energy was calculated for over 4,000 highly ranked compounds. For each compound, the docking pose with the lowest docking energy was selected. Hydrogen atoms were then added, and the structure was converted to mol format using OpenBabel v.3.1.0. MM-GBSA calculations for the highly ranked compounds were conducted in an automated workflow (Uni-GBSA) with default parameters (Yang et al., 2023). Compounds with a binding free energy < −53 kcal/mol were prioritized for further analysis as prioritized hit compounds against AflG.

### 2.5 MD simulations of AflG complex with hit compounds

Of the compounds classified as hits, eight were commercially available. MD simulations using Uni-GBSA and GROMACS 2020 were performed using the top-ranked docking poses of these compounds. The protein was described with the amber99sb force field, while the compounds were described with gaff2. Both were done using default parameters in Uni-GBSA. The simulation parameters were similar to those used for the AflG-Heme-AVN system, except the temperature was maintained at 300 K, and the threshold values for electrostatic and van der Waals interactions were 1.0 nm. Simulations for all systems were run for 100 ns, and trajectories were analyzed as described above. Cluster analysis were conducted for all AflG-compound complexes with the Gromos algorithm and a backbone RMSD threshold of 0.2 nm.

### 2.6 *In vitro* validation of AF biosynthesis inhibition

The *A. flavus* strain NRRL 3357 was used to validate AF biosynthesis inhibition using the previously established liquid incubation system in our lab (Yan et al., 2015; Wu et al., 2022). The eight commercially available candidate inhibitor compounds, identified by PubChem Compound Identifications (CIDs) 50782408, 53209539, 57336812, 53151533, 20880420, 50748540, 91904139, and 54761306, were procured from TopScience Co. Ltd. (Shanghai, China) and had >95% purity. Stock solutions of each compound were prepared in dimethyl sulfoxide (DMSO), except compound 54761306, which was prepared in liquid glucose mineral salt (GMS) medium (Yan et al., 2012). To analyze *A. flavus* physiology and AF production under control (CK) conditions and in the presence of each putative inhibitor, flasks were prepared with 18 mL of liquid GMS medium, 2 mL of spore suspension (0.8 × 10^7^ spores/ml), and 100 μL of putative inhibitor stock solution, DMSO, or GMS medium, yielding an initial fungal density of 0.8 × 10^6^ spores/mL, and the final concentrations of eight putative inhibitors 50782408, 53209539, 57336812, 53151533, 20880420, 50748540, 91904139, and 54761306 were 80, 148, 96, 30, 147, 167, 167, and 48 μM, respectively. All GMS liquid cultures were grown at 28°C under continuous darkness with shaking at 180 rpm (Yan et al., 2012, 2015). The *A. parasiticus* NRRL 2999 strain, obtained from USDA-ARS, was further used to validate the inhibitory effect of compound 50782408 using the same procedure as for the *A. flavus* NRRL 3357 strain. AF contents were measured using thin-layer chromatography (TLC) as previously described (Yan et al., 2012). The relative intensities on the TLC plates were quantified using GelAnalyzer 19.1 (www.gelanalyzer.com) and subsequently normalized to a maximum sample intensity of 1, allowing for the comparison of relative concentrations of AF.

## 3 Results

### 3.1 Atomic-level architecture of AflG and the cofactor-substrate complex

**Figure 1:**
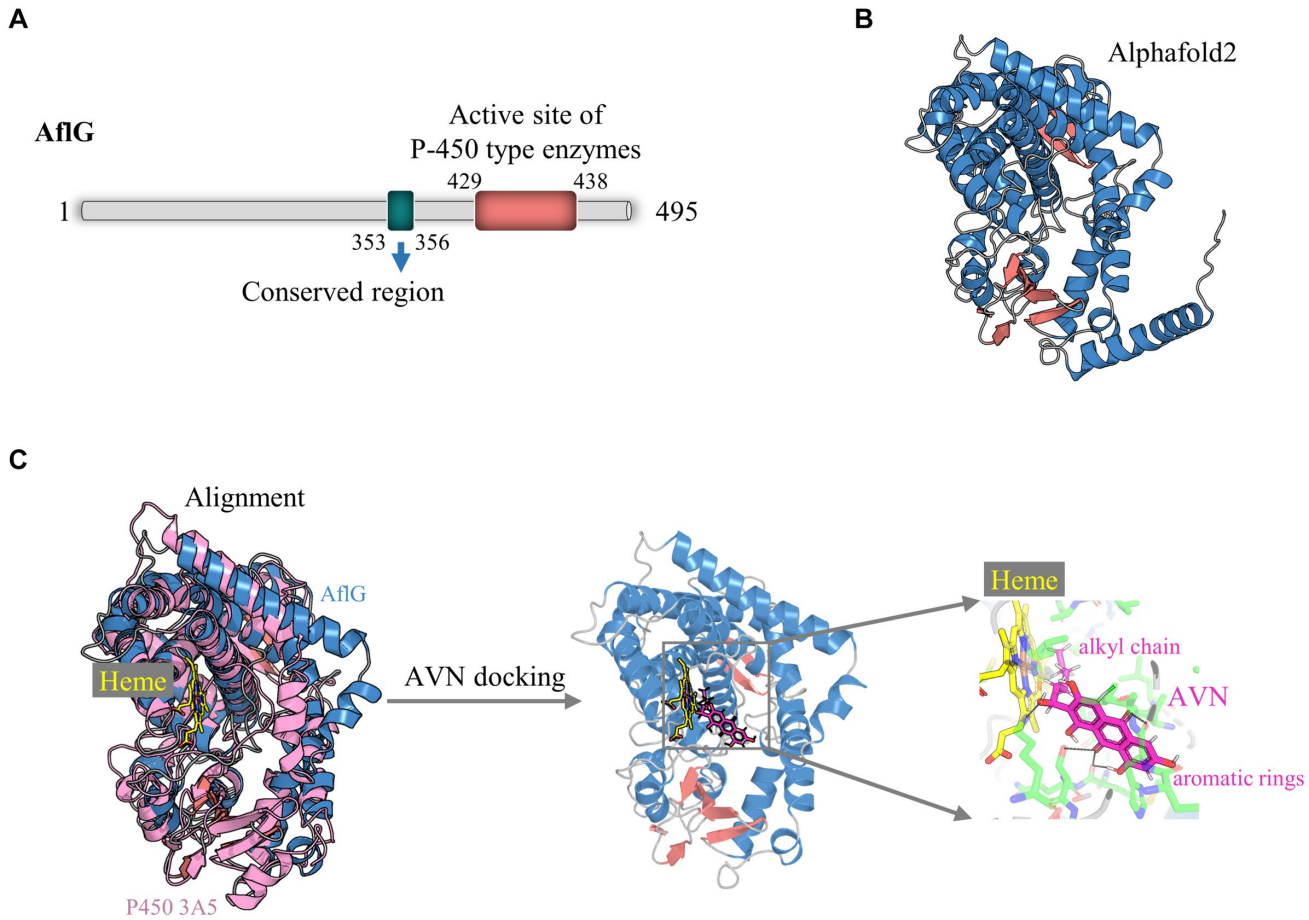
Structural modeling of AflG and the AflG-Heme-averantin (AVN) complex. **(A)** Schematic diagram of the AflG amino acid sequence with conserved cytochrome P450 motifs highlighted. **(B)** AflG structure as predicted with AlphaFold2. Secondary structures are shown: α-helices (blue), β-sheets (red), and loops (gray). **(C)** Structural model of the AflG-Heme-AVN complex. The model was generated by the superposition of AflG and human P450 3A5 complexed with Heme, followed by simulated AVN docking.

Comprehensive structural modeling was undertaken to elucidate the key architectural features of AflG and to provide details of its cofactor and substrate interactions. Bioinformatics analysis revealed that the full-length AflG amino acid sequence (450 residues) contained a highly conserved ExxR motif (E^353^SLR^356^) and an active-site motif (F^429^SIGPRNCIG^438^), both of which are characteristic of cytochrome P450 (Figure 1A) (Yu et al., 1997; Yang et al., 2008; Syed and Mashele, 2014). These findings validated the initial classification of AflG as a member of the cytochrome P450 superfamily. AlphaFold2 was next used to generate a high-confidence three-dimensional AflG structure. This demonstrated that the secondary structure was primarily composed of α-helices (~70%) (Figure 1B), consistent with other well-characterized P450 enzymes. High-confidence pLDDT scores (> 90) for a significant portion of the structure indicated the accuracy of the AflG structural model (Supplementary Figure S1).

In this study, our primary focus was directed toward elucidating the essential residues of AflG that govern its interactions with the substrate, as these aspects are pivotal for understanding the enzymatic function of AflG and facilitating inhibitor screening. Consequently, we omitted the N-terminal 35 amino acids, which were predicted to interact with membranes, from the structural model (Kufareva et al., 2014). Subsequently, we aligned the obtained AflG structure with a closely related human enzyme, P450 3A5, to uncover the binding mode and position of the Heme cofactor (Waterhouse et al., 2018). The superposition of the two structures revealed a close alignment of the Heme-binding α-helical regions (Figure 1C). We, therefore, transferred the Heme position from P450 3A5 to the AflG structure to generate an AflG-Heme complex model. Cavity analysis of the AflG-Heme model demonstrated the presence of a suitable internal binding pocket proximal to the Heme iron center (Supplementary Figure S2) (Gay et al., 2010). The native AflG substrate AVN was then computationally docked into this pocket. A high-scoring pose that positioned the substrate near the Heme with ideal geometry for P450-mediated catalysis was selected as the final AflG-Heme-AVN complex model (Figure 1C) (Lee et al., 2003). Overall, this modeling exercise provided unprecedented atomic-level insights into AflG architecture and the cofactor-substrate complex, enabling further analyses.

### 3.2 MD simulations revealed AflG dynamics and distinct substrate conformational states

**Figure 2:**
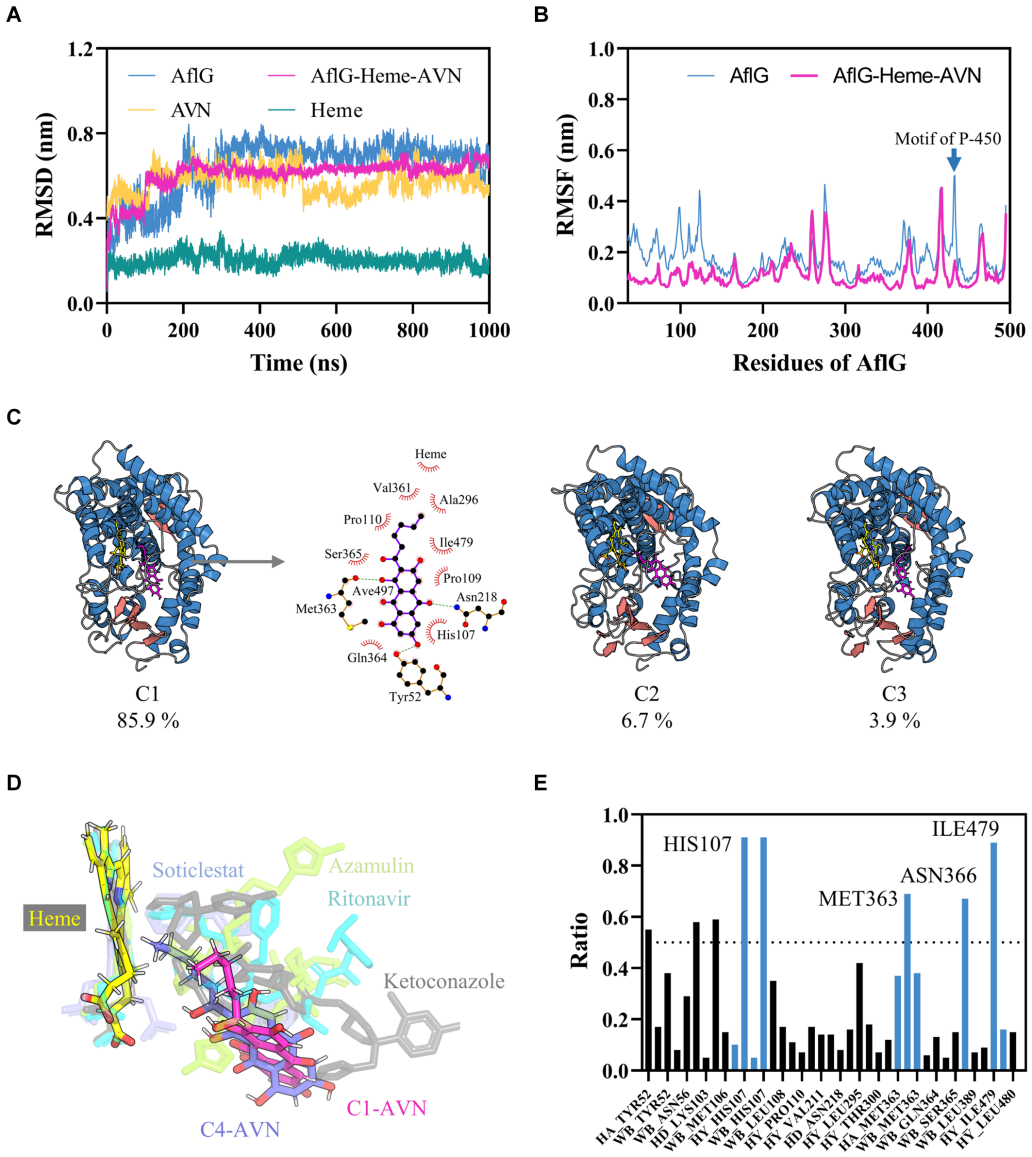
Characterization of interactions between AflG and averantin (AVN) by molecular dynamics simulations. **(A)** Root mean square deviation (RMSD) analysis of AflG and ligands over 1-μs simulations of AflG alone and of the AflG-Heme-AVN complex. **(B)** Root mean square fluctuation (RMSF) values showing AflG residue flexibility with and without AVN bound. **(C)** Representative structures from the top three conformational clusters of the AflG-Heme-AVN complex ensemble, along with two-dimensional interaction diagram illustrating the substrate’s interactions with the protein and Heme. **(D)** Geometric configuration of the Heme cofactor and substrates in the AflG complex compared to related cytochrome P450 structures. **(E)** Ratios of simulation times for specific residue interactions between AflG and AVN.

To gain insights into the conformational dynamics of AflG and its interaction with the substrate AVN, MD simulations at the microsecond timescale were performed on both AflG alone and the AflG-Heme-AVN ternary complex. Equilibration and stability analysis using RMSD measurements revealed that AflG and the AflG-Heme-AVN complex reached equilibrium after approximately 200 ns (Figure 2A). The RMSD of the Heme cofactor remained consistently low throughout each simulation, indicating stable Heme binding and validating the accuracy of the Heme placement. There were periodic fluctuations in the AVN RMSD as the simulations progressed, suggesting that the substrate held various conformational states within the binding pocket. AflG had smaller, more stable RMSD values in the context of the AflG-Heme-AVN complex compared to AflG alone, suggesting that substrate and cofactor binding had a stabilizing effect on AflG. RMSF analysis was conducted to further probe residue flexibility in AflG. This showed that several regions of AflG (namely the N-terminus, residues 260–280, and loops surrounding the active site) were highly flexible in the apo system. In comparison, these regions displayed significantly reduced flexibility in the AflG-Heme-AVN complex (Figure 2B), confirming the stabilizing effects of the substrate and cofactor binding.

In-depth conformational analysis via clustering of the simulation ensembles revealed that the most populated structural clusters exhibited relatively low structural heterogeneity for AflG in both systems (Figure 2C; Supplementary Figure S3). However, more significant N-terminal fluctuations were evident in AflG alone (Figure 2B). As suggested by the RMSD profiles, the representative structures of the most populated clusters in the AflG-Heme-AVN complex clearly showed the AVN substrate shifting between dramatically different conformational states within the binding pocket, with the alkyl chain periodically transitioning between proximal and distal poses with respect to the Heme cofactor (Figure 2C). The two-dimensional interaction diagram of the representative structure from cluster C1 of the AflG-Heme-AVN complex illustrates the interaction of several AflG residues with AVN through hydrogen bond and hydrophobic interactions (Figure 2C). Representative AVN conformations were structurally aligned to crystal structures of other P450 substrate complexes to validate the distinct substrate-binding poses. The simulated AVN conformations were positioned highly similar to known crystal structures of bound ligands in other P450 systems (Figure 2D), indicating the validity of the predicted substrate binding locations in this context.

An interaction fingerprint analysis was then conducted to identify critical intermolecular interactions between AflG and the AVN substrate over the course of the simulations (Kokh et al., 2020). This revealed eight specific interactions that recurred with a contact ratio exceeding 50% of the simulation time. These interactions included hydrogen bonds, hydrophobic contacts, and water bridges. Notably, the AflG residues His107, Met363, Asn366, and Ile479 interacted with AVN during >65% of the simulation time (Figure 2E), highlighting these residues as potentially crucial contact points for substrate binding.

### 3.3 Large-scale virtual screening yielded potential hit compounds against AflG

**Figure 3:**
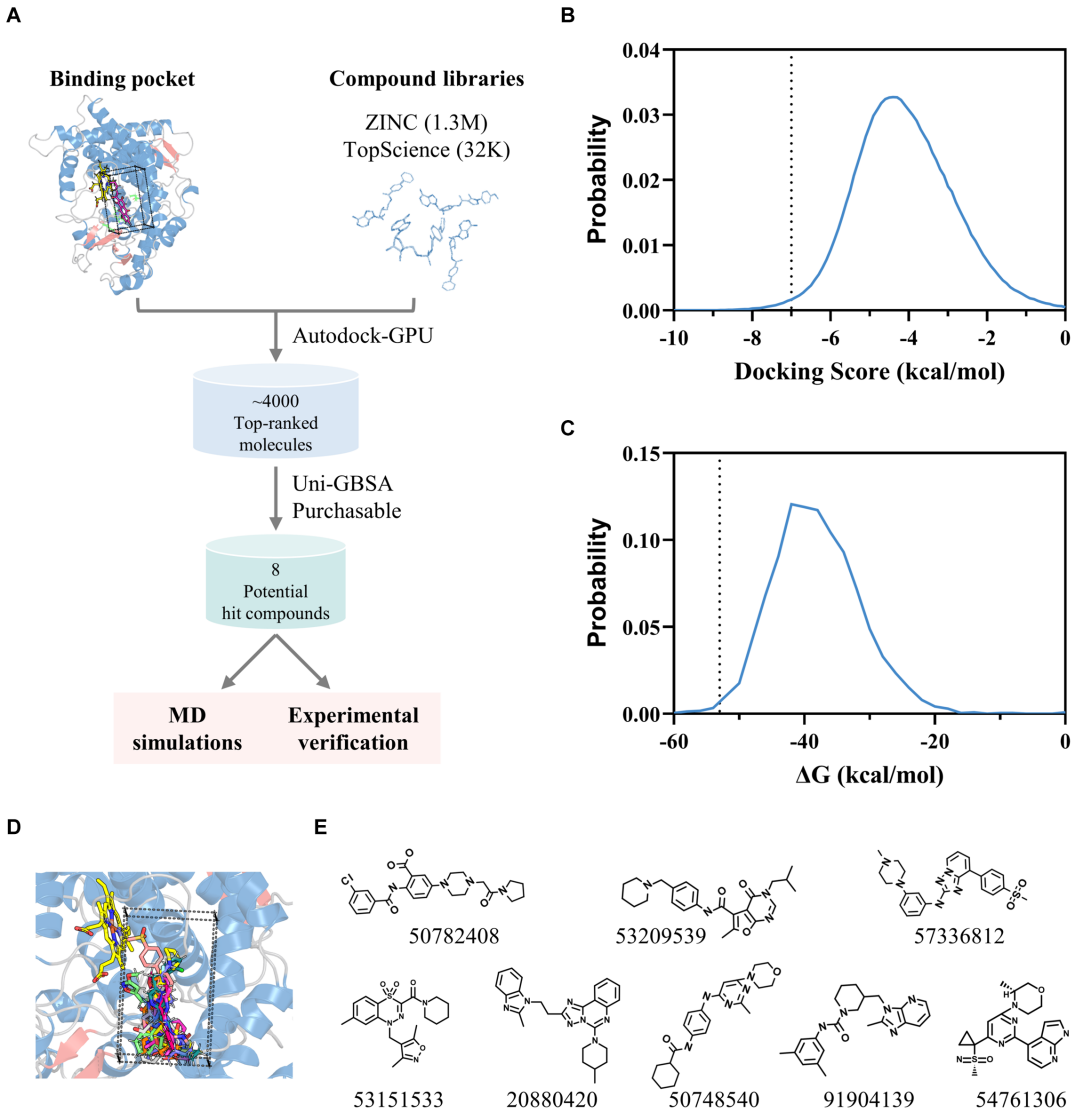
Identification of potential hit compounds against AflG with structure-based virtual screening and MM-GBSA calculations. **(A)** Virtual screening workflow. **(B)** Distribution of docking scores for all screened compounds. The dashed line indicates the threshold (−7 kcal/mol). **(C)** Distribution of calculated binding free energies for highly ranked compounds from the docking simulation. The dashed line indicates the threshold (−53 kcal/mol) for further analysis. **(D)** Superposition of screened compounds with the most favorable binding free energies. **(E)** Molecular diagrams of the eight commercially available top-ranked compounds selected as potential hit compounds against AflG, corresponding labeled by PubChem CID numbers, are shown below each compound.

Leveraging the AflG-AVN structural model and the identified key binding residues, we next conducted a systematic, structure-based virtual screening to identify potential hit compounds against AflG (Figure 3A). Using two separate compound libraries, we screened over 1.3 million molecules from the ZINC20 database and an additional 32,000 molecules. This comprehensive screening yielded more than 4,000 top-ranked compounds with favorable docking scores (< −7 kcal/mol) (Figure 3B), indicating significant predicted binding affinity. Free energy calculations were next conducted for the top-ranked compounds using MM-GBSA. This revealed 15 compounds with remarkably low binding free energies (< −53 kcal/mol) (Figure 3C; Table 1), which were selected as hits for further analyses.

**Table 1:** Docking scores and binding free energies of averantin (AVN) and the potential hit compounds against AflG from virtual screening and MM-GBSA calculations.

Compound	Docking score (kcal/mol)	Binding free energy (kcal/mol)
AVN	−5.88	−47.50
50782408	−7.12	−60.25
92857748	−7.2	−58.47
53209539	−7.36	−58.31
95894266	−7.12	−58.29
57336812	−8.79	−56.25
124366745	−7.04	−55.79
53151533	−8.06	−55.46
20880420	−9.16	−55.30
92408135	−7.85	−54.80
124366746	−7.17	−54.65
124893813	−7.07	−54.39
135404553	−7.1	−54.31
50748540	−8.39	−54.14
91904139	−8.28	−53.27
54761306	−7.09	−53.02

AVN is the native substrate. Other compounds are identified by their PubChem CID numbers.

Structural analysis indicated that these 15 compounds consistently occupied a highly similar position within the AVN substrate binding pocket (Figure 3D). Eight of the compounds were commercially available and therefore prioritized for validation (Figure 3E).

### 3.4 MD simulations verified the binding stability of prioritized hit compounds

**Figure 4:**
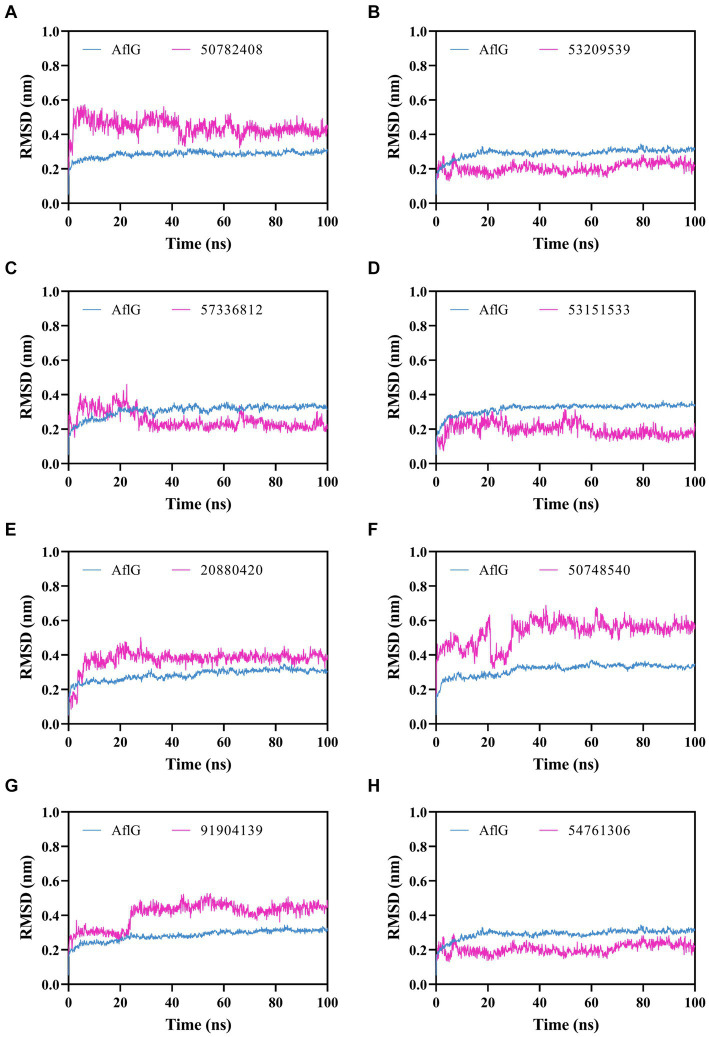
Molecular dynamics simulations of AflG bound to prioritized hit compounds. RMSD values of AflG (blue) and ligand (purple) in complex are shown over the course of the 100-ns simulation time for compounds **(A)** 50782408, **(B)** 53209539, **(C)** 57336812, **(D)** 53151533, **(E)** 20880420, **(F)** 50748540, **(G)** 91904139, and **(H)** 54761306.

To characterize interactions between AflG and the prioritized hit compounds, 100-ns MD simulations were performed for each AflG-hit complex. AflG RMSD plots revealed minimal structural deviations (~0.2 nm) across all of the simulations (Figure 4), indicating that the binding of each compound did not dramatically alter the overall AflG conformation compared to AflG in the AVN-bound state. Ligand RMSD measurements showed similarly small deviations in compounds 53209539, 57336812, 53151533, and 54761306, suggesting that these compounds were stably bound (Figures 4B–D, H). Compounds 50782408, 20880420, 50748540, and 91904139 displayed larger RMSD fluctuations, indicating significant changes in their poses from the initial docked conformations (Figures 4A,E–G).

**Figure 5:**
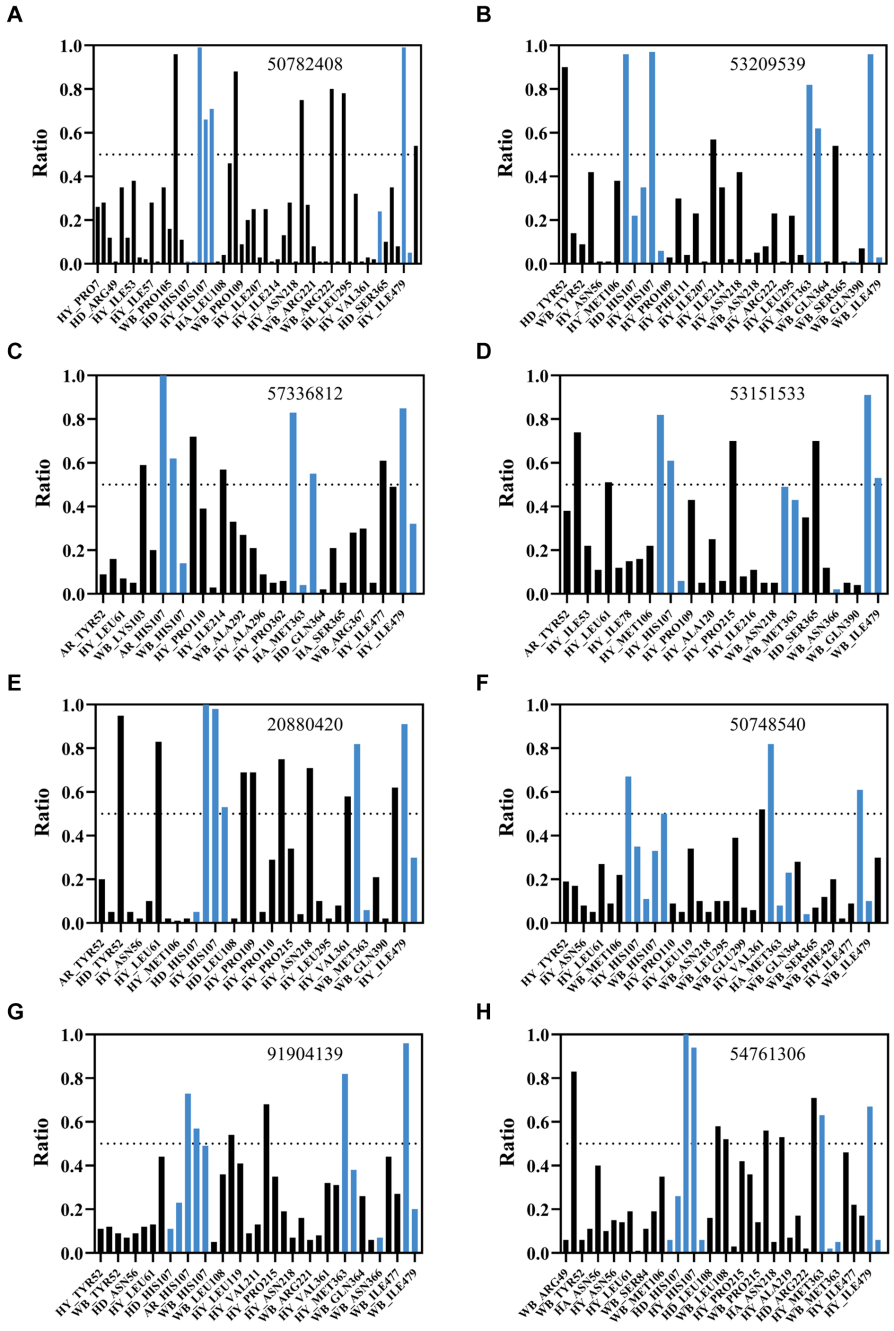
Analysis of interactions between AflG and prioritized hit compounds. The ratios of specific residue interaction times were analyzed between AflG and compounds **(A)** 50782408, **(B)** 53209539, **(C)** 57336812, **(D)** 53151533, **(E)** 20880420, **(F)** 50748540, **(G)** 91904139, and **(H)** 54761306. The Y-axis represents the proportion of interaction time to the total simulation time. The X-axis represents different interaction types, including water bridge (WB), hydrophobic interaction (HY), hydrogen bond acceptor (HA), and hydrogen bond donor (HD), involving different residues.

Binding free energy calculations for representative cluster structures showed improved affinity compared to the docked poses for all compounds (Supplementary Table S1), confirming that each compound was predicted to bind to AflG with a much stronger affinity than the native substrate (AVN). Interaction fingerprint analysis revealed key sustained contacts of most of the compounds with the AflG residues His107, Met363, and Ile479 (Figure 5; Table 2). Compounds 50782408 and 20880420 showed more persistent interactions within the binding pocket than AVN did, supporting the validity of the predicted competitive binding modes. However, compounds 50748540 and 91904139 showed fewer contact points compared to AVN. Overall, these MD simulations verified the stable binding of several high-priority hit compounds to AflG, adopting binding modes similar to that of the native substrate AVN, indicating their potential as AflG inhibitors.

**Table 2:** Key AflG residues predicted to interact with ligands.

Ligand	Binding free energy (kcal/mole)	Hydrogen-bond	Hydrophobic interaction	Water bridge	Aromatic interaction	Number of interactions
AVN	−47.50	Tyr52	His107, Ile479, Met363	Asn366, Glu82, His107, Lys103		8
50782408	−60.25	Met106	His107, Ile479, Leu480, Pro109, Pro7	Asn218, His107, Tyr223	His107	10
53209539	−58.31	Ser365, Tyr52	His107, Ile479, Met363, Val211	Met106, Met363	His107	9
57336812	−56.25		His107, Ile214, Ile477, Ile479, Met363, Pro109	Lys103, Met363	His107	9
53151533	−55.46	Ser365	His107, Ile479, Leu61, Pro215, Tyr52	Ile479	His107	8
20880420	−55.30	Tyr52	Asn218, His107, Ile214, Ile477, Ile479, Leu61, Met363, Pro109, Val361	His107, Leu108	His107	13
50748540	−54.14	His107	Ile479, Met363, Pro109, Val361			6
91904139	−53.27		His107, Ile214, Ile479, Met363, Pro109		His107	6
54761306	−53.02	Leu108	Asn218, His107, Ile479, Met363, Tyr52	Asn218, Leu108	His107	9

AflG residues included here showed intermolecular contacts with the indicated compound for >50% of the molecular dynamics simulation time. Averantin (AVN) is the native substrate. Other compounds are identified by their PubChem CID numbers.

### 3.5 Experimental validation of putative AflG inhibitors

**Figure 6:**
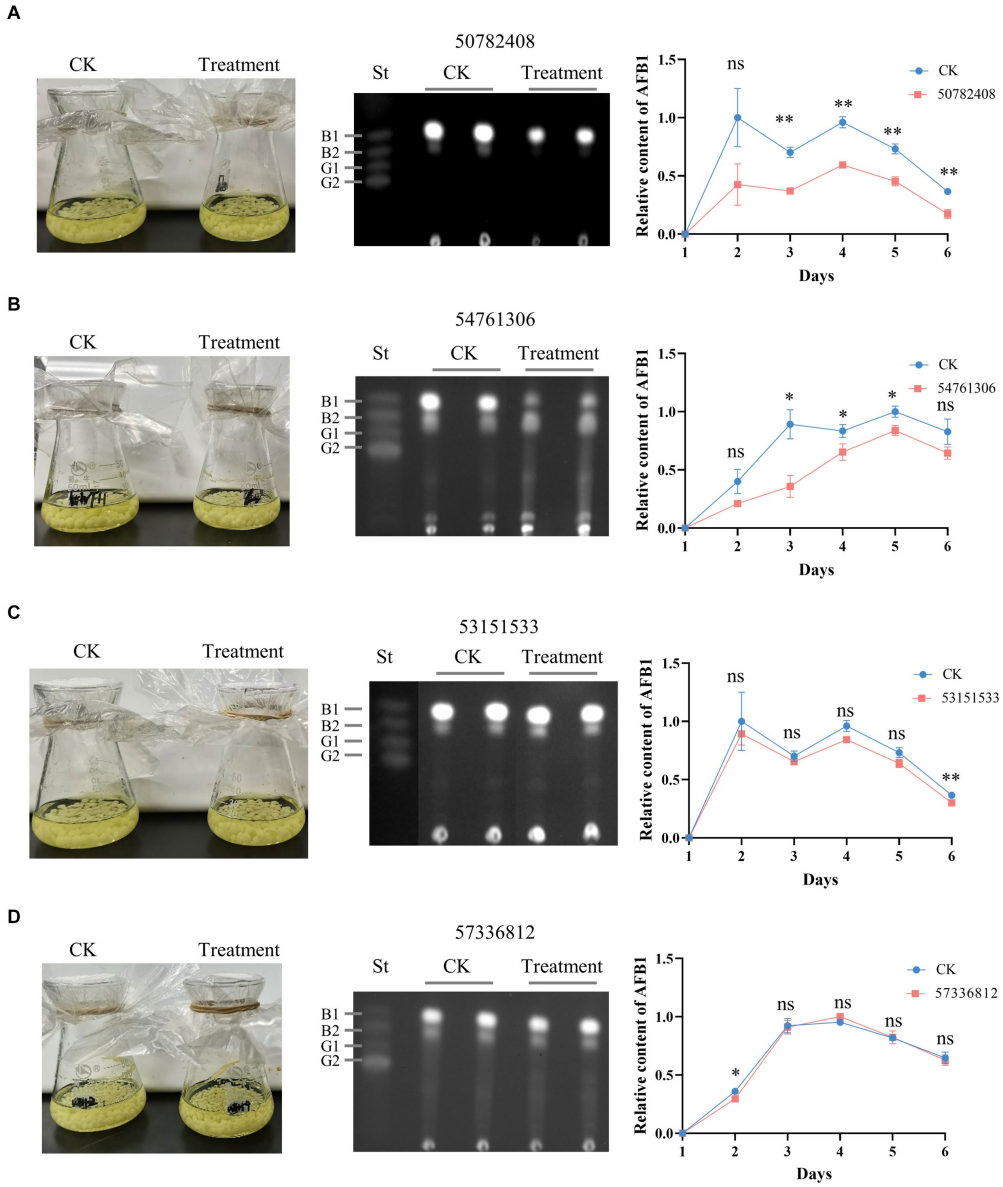
Effects of candidate AflG inhibitors on aflatoxin (AF) biosynthesis in *Aspergillus flavus*. **(A–D)** *A. flavus* control cultures (CK) and *A. flavus* cultures treated with the compounds, TLC of extracted AFs, and quantified relative intensity of AF production in *A. flavus* treated with compounds **(A)** 50782408, **(B)** 54761306, **(C)** 53151533, or **(D)** 57336812. Left, culture appearance after growth for 3-d. Center, visualization of extracted AF via thin-layer chromatography (TLC) from 3-d cultures, where ‘St’ represents aflatoxin standards. Right, relative content of AF quantified from TLC plates. The figures show the experimental results from two independent biological replicates. Error bars indicate the standard deviation (St) of the measurements. Significance was determined by the *t*-test. *p < 0.05, **p < 0.01, ***p < 0.001, ****p < 0.0001.

The effects of putative AflG-inhibitory compounds on AF biosynthesis were explored experimentally using *A. flavus* strain NRRL 3357. Mycelial phenotypes and AF levels in the growth medium were monitored over 6 days in CK cultures and those with selected compounds added. There were no significant differences in mycelial phenotypes between any of the treated cultures and the CK (Figure 6), demonstrating a lack of toxicity to the fungus. However, compounds 50782408 and 54761306 notably inhibited AF production (Figures 6A,B). Specifically, compound 50782408 significantly inhibited AF production over the entire culture time (Figure 6A), whereas compound 54761306 had a remarkable effect on the third day of growth (Figure 6B). Contrary to our expectations based on the predictions, the remaining six compounds (53151533, 57336812, 53209539, 20880420, 50748540, and 91904139) had no measurable inhibitory impacts on mycelial growth or AF production (Figures 6C,D; Supplementary Figure S4).

Given the promising inhibitory effects of compound 50782408 on AF biosynthesis observed in the above experiments, we further conducted a concentration titration experiment to validate its efficacy. We observed a decrease in AF production with increasing concentrations of compound 50782408 (Supplementary Figures S5, S6). Additionally, we treated another aflatoxigenic strain, *A. parasiticus* NRRL 2999, with compound 50782408. The results showed that, consistent with observations in *A. flavus* NRRL 3357, compound 50782408 exhibited a significant inhibitory effect on the strain *A. parasiticus* NRRL 2999, affirming its potent inhibitory action (Supplementary Figure S7). Collectively, the experimental validations of candidate AflG inhibitors pinpointed two compounds that inhibit AF biosynthesis, consistent with our computational predictions.

### 3.6 Structural and dynamic analyses revealed the distinct binding modes underlying functional inhibitory effects

**Figure 7:**
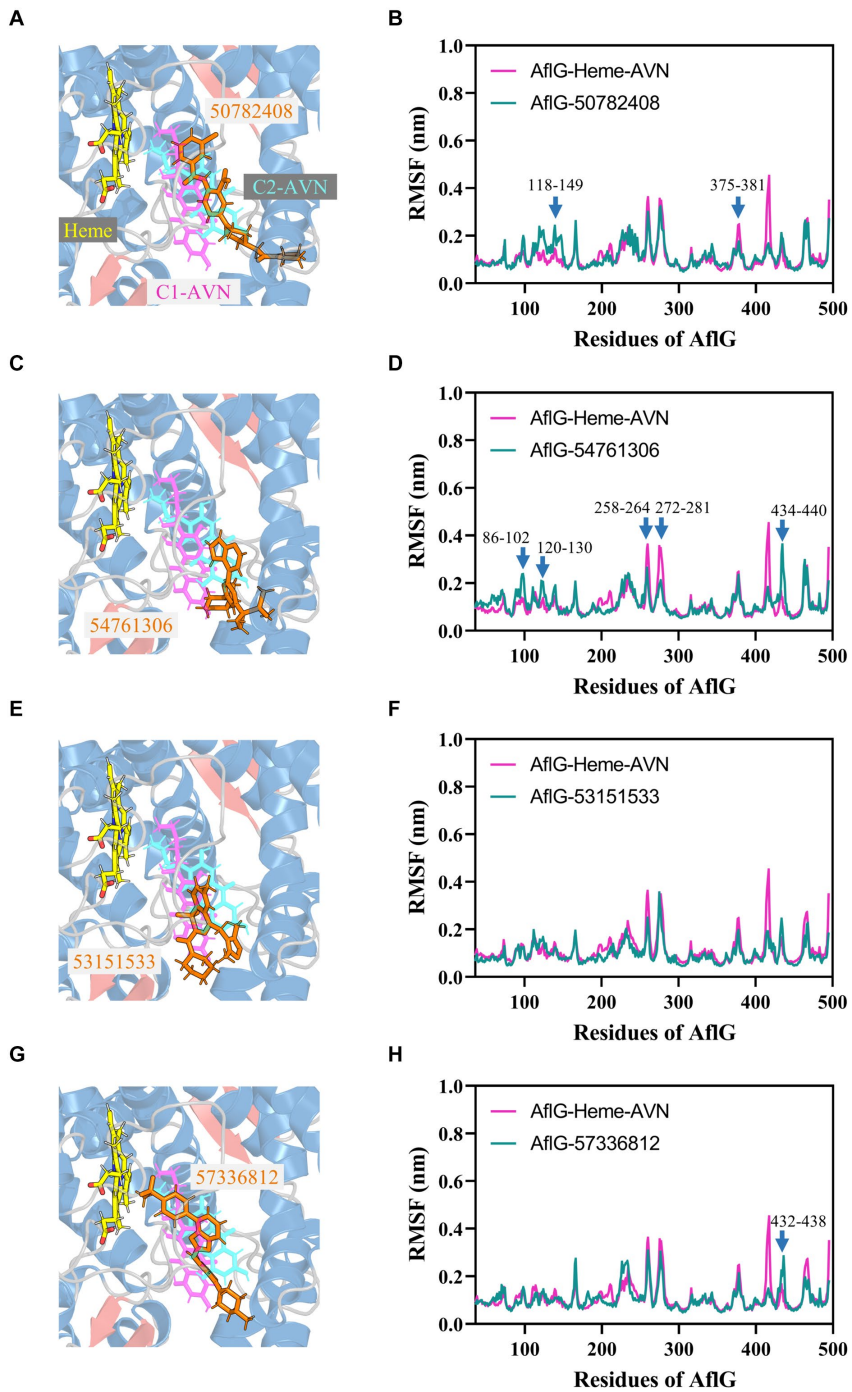
Binding modes of putative AflG inhibitors and their effects on AflG flexibility. **(A,C,E,G)** AflG-ligand complex structures showing the binding orientations of compounds **(A)** 50782408, **(C)** 54761306, **(E)** 53151533, and **(G)** 57336812. Each candidate inhibitor compound structure is overlaid with the substrate averantin (AVN) in the AflG-Heme-AVN complex. **(B,D,F,H)** Root mean square fluctuation (RMSF) plots comparing fluctuations in AflG residues between the AflG-Heme-AVN complex and AflG complex with compounds **(B)** 50782408, **(D)** 54761306, **(F)** 53151533, and **(H)** 57336812.

Structural comparisons and dynamics analyses were undertaken to elucidate the molecular mechanisms underlying the distinct effects of candidate compounds on AF biosynthesis. Structural alignment of the top cluster for each AflG-compound ensemble revealed that the active inhibitor 50782408 occupied the AflG binding pocket comparably to the native substrate AVN (Figure 7A). Meanwhile, the binding location of compound 54761306 was comparable to that of the AVN aromatic rings only, despite the inhibitory activity of compound 54761306 (Figure 7C). The inactive compound 53151533 demonstrated a comparable proximal localization to the aromatic core of AVN but lacked functional efficacy (Figure 7E). Additionally, the inactive compound 57336812 displayed a similar proximity to AVN but had no impact on AF production (Figure 7G).

Dynamic analyses based on RMSF values indicated that interactions with most of the candidate inhibitor compounds enhanced AflG rigidity and stability, consistent with enzyme characteristics in an inhibitor-bound state. Specifically, interactions with compounds 50782408 and 91904139 increased the flexibility of the AflG N-terminal region compared to the substrate AVN (Figure 7B; Supplementary Figure S8D). Other compounds (e.g., 54761306, 53209539, and 20880420) had a milder impact on AflG N-terminal dynamics (Figure 7D; Supplementary Figure S8). Several compounds, including 54761306, 57336812, 20880420, 53151533, and 50748540, also increased movement dynamics of the active site motif (residue 426–442), whereas only compounds 50782408 and 50748540 reduced flexibility within the 375–381 loop near the active site (Figure 7B, F, H; Supplementary Figure S8C). Despite the adoption of unique binding poses by each compound, these analyses thus revealed that compounds 50782408 and 54761306 inhibited AF biosynthesis while subtly modulating AflG dynamics through localized stabilization or destabilization of specific regulatory regions. Despite affecting similar dynamic changes and engaging in numerous intermolecular contacts with AflG, the ineffectiveness of compound 20880420 as an AflG inhibitor highlights the complexity of correlating interactions and protein dynamics with downstream functional impacts (Supplementary Figure S8B; Table 2). Further biophysical and structural studies are essential to increase our understanding of the ways in which interactions with specific compounds modulate AflG structure and dynamics and inhibit AF biosynthesis.

## 4 Discussion

Crop contamination with AFs impacts both the food and feed supply chains, exposing humans and livestock to these harmful compounds (Caceres et al., 2020; Uka et al., 2020). Such exposure not only jeopardizes human health but also induces substantial economic losses across the world. It is thus imperative to devise strategies that curtail AF biosynthesis in agricultural products. Enzyme inhibitors, which can be applied to regulate metabolic activity, offer a potential method for achieving this goal (Tabe et al., 2019; Manzoor and Hoda, 2020; Sharma et al., 2020; Labib et al., 2022). Identification of inhibitors that target critical enzymes in the AF biosynthetic pathway is a promising strategy for minimizing AF contamination.

In the present study, we harnessed an integrated computational-experimental strategy, incorporating structural modeling, MD simulations, virtual screening, binding free energy calculations, and *in vitro* experimental validation to explore the structure, dynamics, and potential inhibition mechanisms of the critical AF biosynthetic enzyme AflG. Advanced structural prediction and modeling techniques enabled the construction of highly accurate AflG models, both of the enzyme alone and in complex with its native substrate (Kufareva et al., 2014; Waterhouse et al., 2018; The UniProt Consortium, 2023). Microsecond-scale MD simulations provided remarkable insights into the conformational landscape and dynamic motions involved in substrate binding (Abraham et al., 2015; Huang et al., 2017). The ligand AVN was observed to hold distinct poses within the binding pocket, periodically transitioning between conformations in which it was proximal and distal to the Heme cofactor. Furthermore, AflG displayed modulated dynamics upon substrate binding, with reduced structural fluctuations, particularly in the N-terminus and the loops surrounding the active site. These simulations demonstrated the power of molecular modeling to capture the inherent flexibility and transient motions governing this molecular system.

Building upon these initial results, we conducted a large-scale virtual screening of over 1.3 million compounds to identify potential hit compounds against AflG (Santos-Martins et al., 2021). Stringent binding free energy calculations yielded 15 high-confidence candidates, and subsequent MD simulations corroborated the robust binding attributes of specific compounds that shared key interaction residues with AVN (namely His107, Met363, and Ile479) (Yang et al., 2023). *In vitro* experiments with *A. flavus* strain NRRL 3357 showed that two of these compounds, 50782408 and 54761306, markedly inhibited AF biosynthesis throughout a 6-d growth period. These results highlight the potential of compounds 50782408 and 54761306 as targeted modulators of the AF metabolic pathway.

Although several of the candidate compounds demonstrated potent inhibition of AF biosynthesis, consistent with the computational predictions, several other candidates proved ineffective *in vitro*, emphasizing the challenges in extrapolating predicted binding affinities to functional outcomes. For example, compound 53151533 was predicted to have a low binding free energy but exhibited no discernible influence on AF levels. Conformational analysis revealed that compounds 50782408 and 54761306, which functionally inhibited AflG, occupied binding regions close to that of the native substrate AVN or its aromatic rings. In contrast, the inactive compound 53151533 was positioned near the binding regions associated with the AVN aromatic rings despite having a similar predicted affinity as those of compounds 50782408 and 54761306. These findings further highlight the complexity of predicting AF production effects from AflG-inhibitor interaction paradigms and binding energy calculations. This discrepancy could stem from limitations of *in silico* modeling in accounting for complex *in vivo* conditions, differences between kinetic and thermodynamic aspects of inhibition, potential compensatory mechanisms in the fungal system, and bioavailability issues affecting the compounds’ ability to reach their target (Xu et al., 2021). To address these challenges, further efforts should prioritize the iterative refinement of structure–activity correlations, leveraging an integrated blend of computational and biophysical methodologies to enhance predictive accuracy continuously (Wu et al., 2020). Future studies could also integrate artificial intelligence (AI) technologies, such as machine learning algorithms, to improve the efficiency and reliability of the screening process (Patel et al., 2020).

Overall, this study establishes a powerful framework, including multi-scale modeling, high-throughput screening, and experimental validation to identify inhibitors of key AF biosynthetic enzymes. We successfully identified several compounds that functionally inhibited AF biosynthesis *in vivo*, and subsequent iterative improvements could be used to screen compounds with more pronounced pesticide-like attributes. Furthermore, the high-resolution models of AflG, both alone and in the substrate-bound form, provide a wealth of information to guide structure-based design. Ultimately, mitigating AF contamination at all stages of agricultural production requires collaborative solutions across the fields of agriculture, toxicology, and public health. Unraveling the biological mechanisms underlying AF biosynthesis is a cornerstone of any approach seeking to minimize AF contamination.

## 5 Conclusion

We here integrated advanced computational modeling approaches with *in vitro* validation of AF production inhibitory activity to explore functional mechanisms and identify inhibitors of a key enzyme in AF biosynthesis. Predictive structural modeling yielded detailed structural insights into the hitherto unknown structure of AflG. Furthermore, MD simulations revealed unique binding conformations in AflG that had remained undiscovered in static structural representations of enzyme-substrate interactions. A high-throughput virtual screening strategy was used to identify hit compounds against AflG, which were then validated *in vitro*. This structure-guided pipeline yielded promising candidate inhibitors, compounds 50782408 and 54761306. Importantly, our approach offered unprecedented insights into the atomistic characteristics of a critical AF biosynthetic enzyme both alone and in complex with substrates, enabling future optimization of efforts to identify compounds to combat AF contamination in agricultural products. The integrated strategy described here could also be employed to unearth putative inhibitors of other mycotoxin biosynthetic pathways, contributing to decreased economic losses and disease burden arising from fungal specialized metabolite contamination in agricultural products.

## Data availability statement

The original contributions presented in the study are included in the article/Supplementary material, further inquiries can be directed to the corresponding authors.

## Author contributions

FW: Data curation, Formal analysis, Investigation, Methodology, Validation, Writing – original draft. WZ: Investigation, Validation, Writing – original draft. MY: Data curation, Methodology, Writing – original draft. JN: Data curation, Writing – original draft. WH: Data curation, Writing – original draft. ZC: Data curation, Writing – original draft. YC: Investigation, Writing – original draft. DW: Methodology, Writing – original draft. JZ: Funding acquisition, Supervision, Writing – original draft. SW: Conceptualization, Data curation, Formal analysis, Funding acquisition, Investigation, Methodology, Writing – original draft. SY: Funding acquisition, Project administration, Resources, Supervision, Writing – review & editing.
