# Supplementary material for: Structure-guided discovery of novel AflG inhibitors for aflatoxin contamination control in aspergillus flavus
Source: Front Microbiol. 2024 Jul 12;15:1425790. doi: 10.3389/fmicb.2024.1425790 (PMC11272468; doi:10.3389/fmicb.2024.1425790)
Supplement: Supplementary file 1 [file Data_Sheet_1.docx]

**Supporting Information**

**Structure-Guided Discovery of Novel AflG Inhibitors for Aflatoxin Contamination Control in *Aspergillus flavus***

Fenghua Wang^a, b^, Weijie Zhou^b^, Maohua Yang^c^, Jinlu Niu^b^, Wenjie Huang^b^, Zhaofu Chen^b^, Yuanyuan Chen^b^, Dongdong Wang^c^, Jun Zhang^a, *^, Shaowen Wu^b, *^, Shijuan Yan^b, *^

^a^ College of Resources and Environmental Sciences, Gansu Agricultural University, Lanzhou, 730070, China

^b^ State Key Laboratory of Swine and Poultry Breeding Industry; Guangdong Key Laboratory for Crop Germplasm Resources Preservation and Utilization, Agro-biological Gene Research Center, Guangdong Academy of Agricultural Sciences, Guangzhou, 510640, China

^c^ DP Technology, Beijing, China

* To whom correspondence may be addressed.

Jun Zhang, E-mail: [zhangjun@gsau.edu.cn](mailto:zhangjun@gsau.edu.cn)

Shaowen Wu, E-mail: [wushaowen@agrogene.ac.cn](mailto:wushaowen@agrogene.ac.cn)

Shijuan Yan, E-mail: [shijuan@agrogene.ac.cn](mailto:shijuan@agrogene.ac.cn)

**Contents**

**Figure S1**. AlphaFold2 predicted structure of AflG with a colored per-residue confidence score (pLDDT).

**Figure S2**. The identified binding pocket of AflG by CavityPlus.

**Figure S3.** Representative structures from the top five conformational clusters of the AflG ensemble.

**Figure S4.** Effects of candidate AflG inhibitors on aflatoxin (AF) biosynthesis in *Aspergillus flavus*.

**Figure S5**. TLC of extracted aflatoxins in *A. flavus* culture treated with different concentrations of compound 50782408.

**Figure S6**. Relative content of aflatoxin B1 in *A. flavus* culture treated with different concentrations of compound 50782408.

**Figure S7.** Effects of compound 50782408 on aflatoxin (AF) biosynthesis in the *Aspergillus parasiticus* NRRL 2999 strain.

**Figure S8.** Effects of hit compounds on AflG flexibility.

**Table S1.** Binding free energies of putative AflG inhibitors from virtual screening


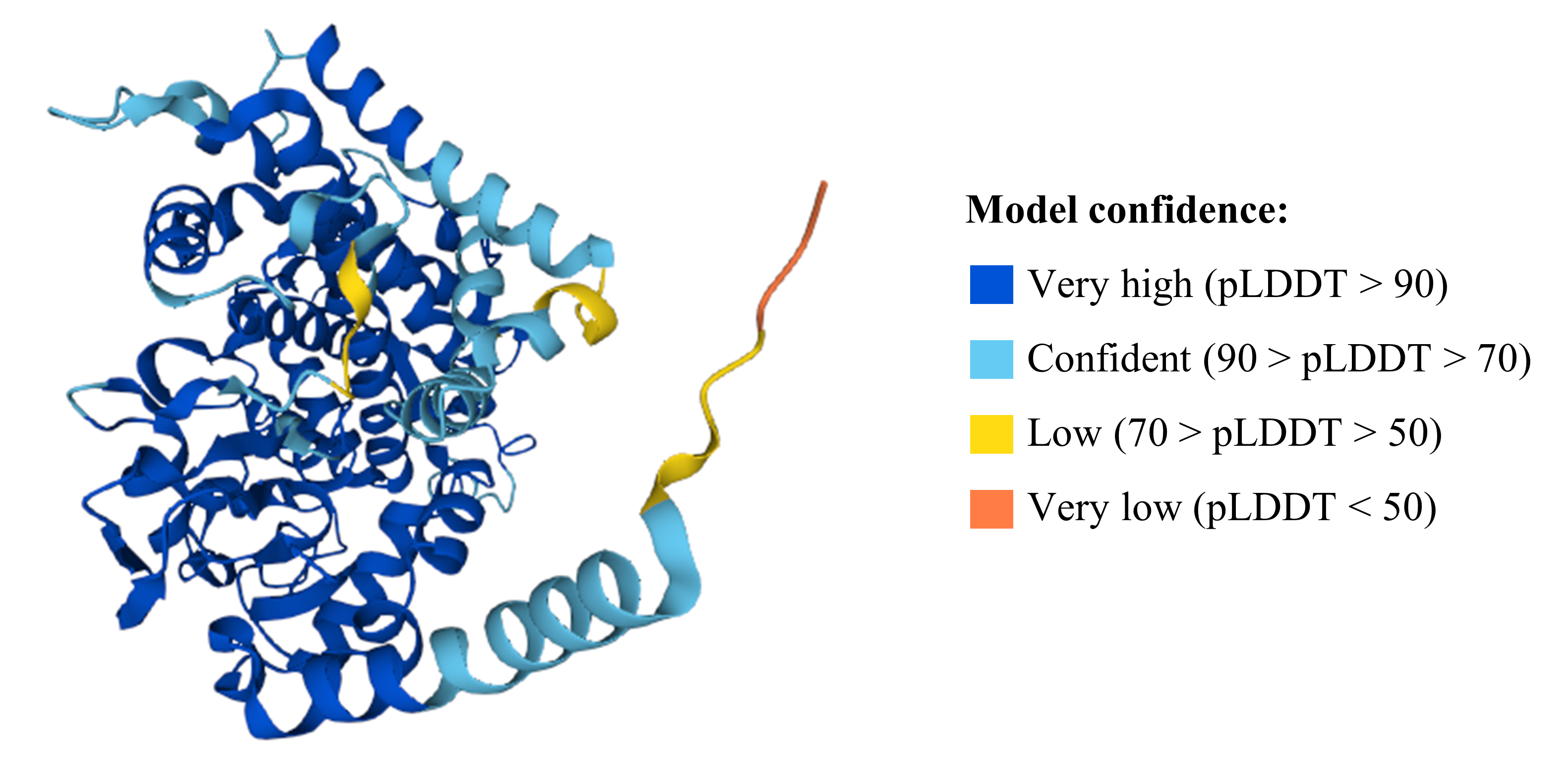


**Figure S1.** AlphaFold2 predicted structure of AflG with a colored per-residue confidence score (pLDDT).


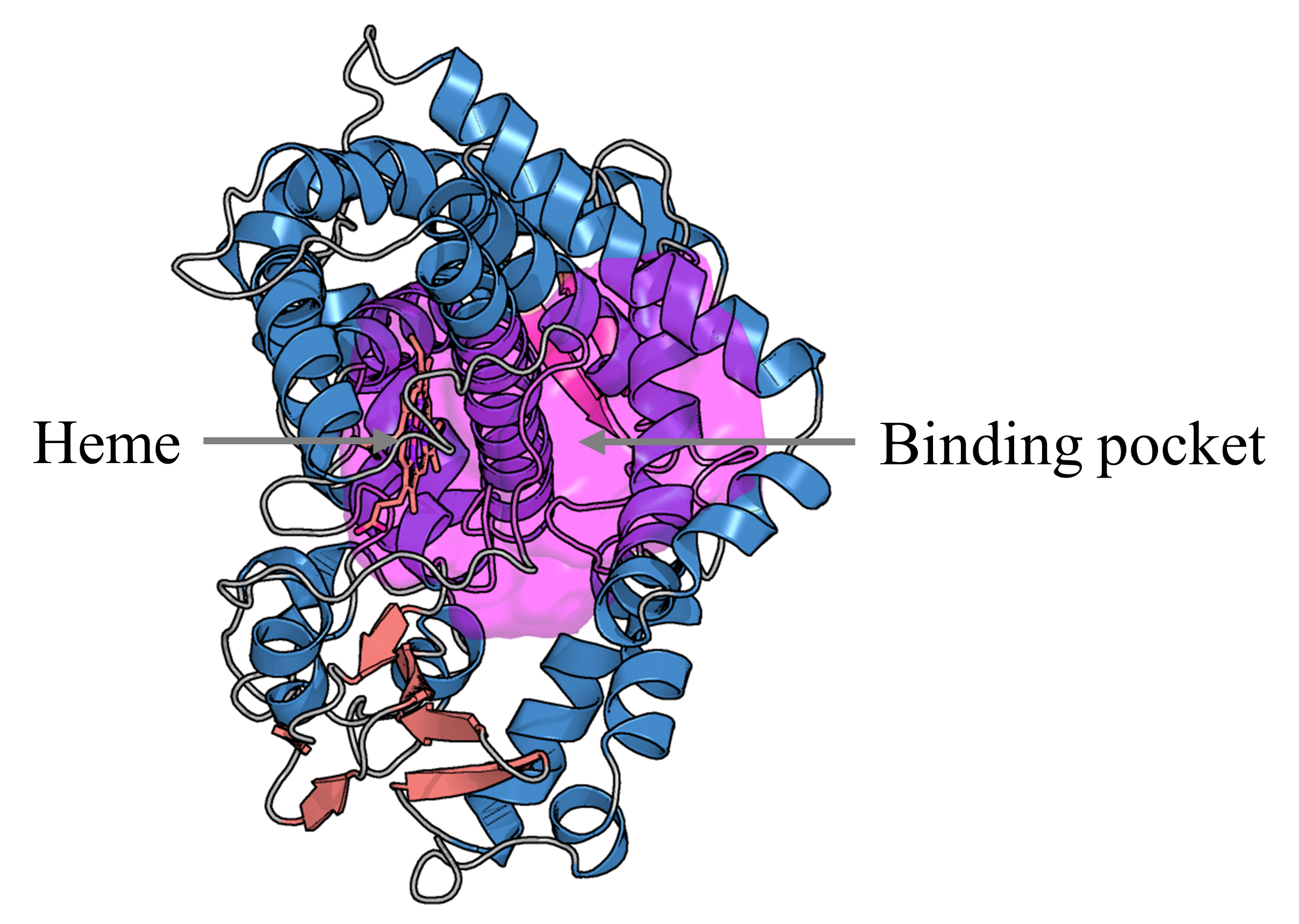


**Figure S2.** The identified binding pocket of AflG by CavityPlus.


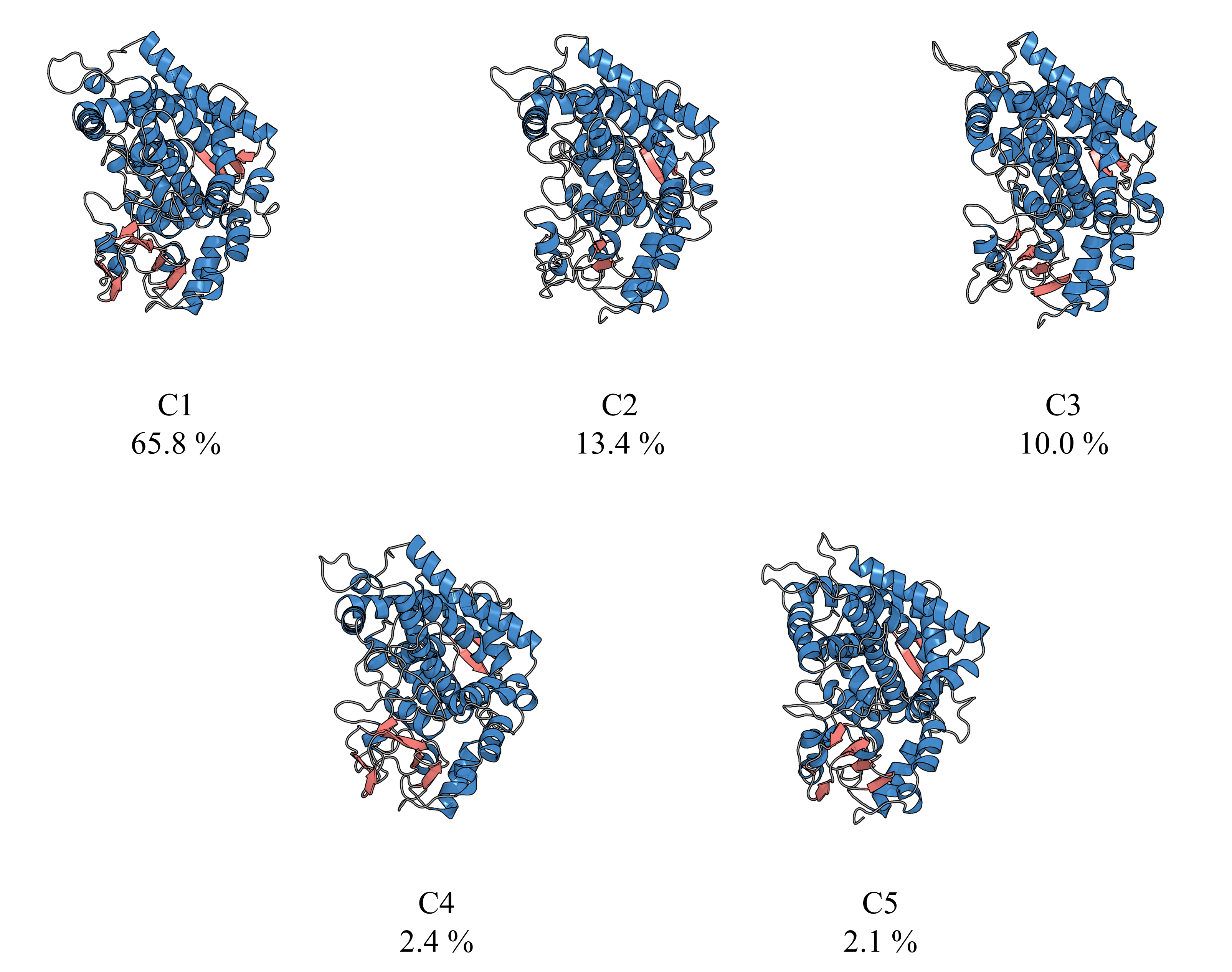


**Figure S3.** Representative structures from the top five conformational clusters of the AflG ensemble.


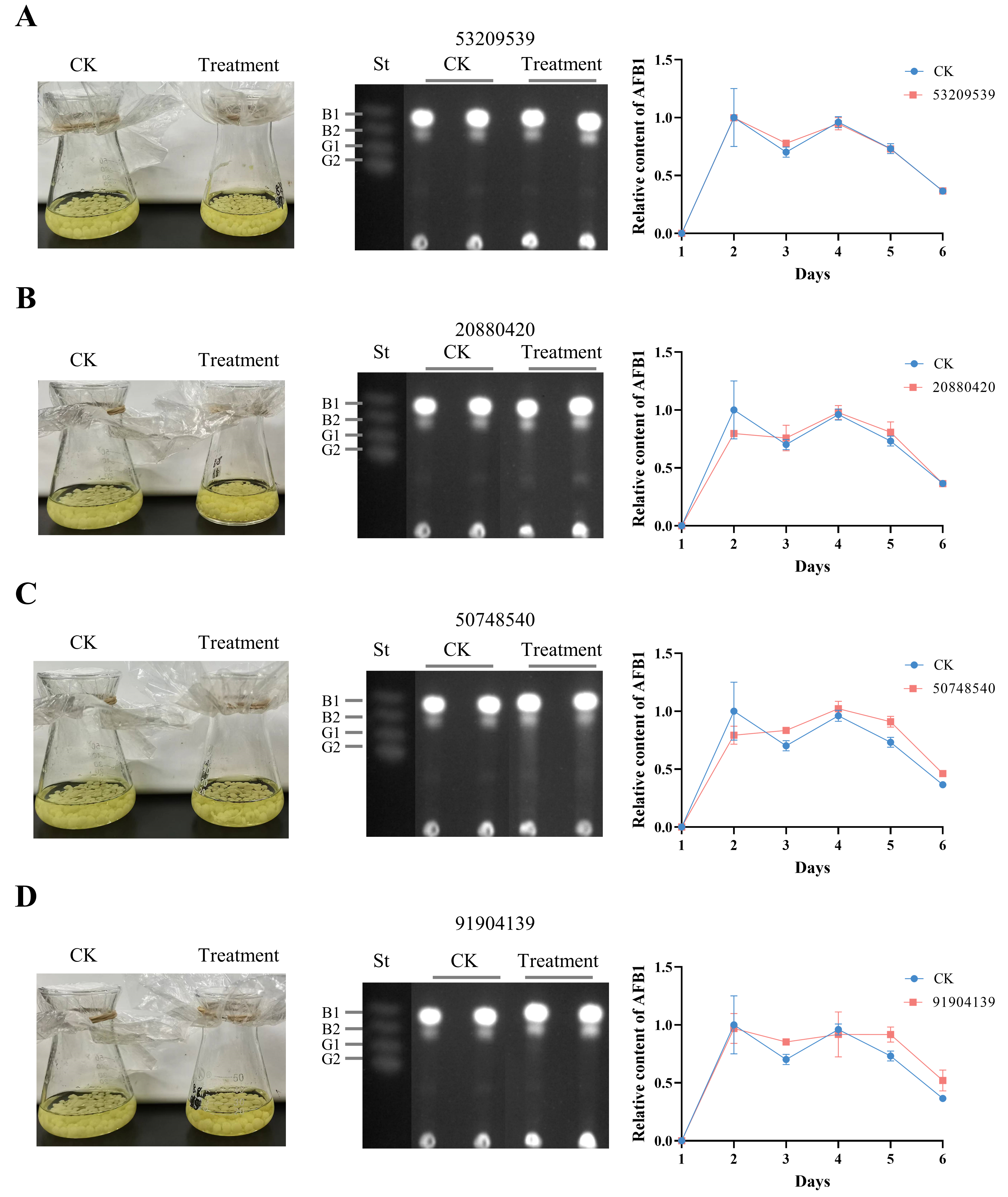


**Figure S4. Effects of candidate AflG inhibitors on aflatoxin (AF) biosynthesis in *Aspergillus flavus*.** (A-D) *A. flavus* control cultures (CK) and *A. flavus* cultures treated with the compounds, TLC of extracted AFs, and quantified relative intensity of AF production in *A. flavus* treated with compounds (A) 53209539, (B) 20880420, (C) 50748540, or (D) 91904139. Left, culture appearance after growth for 3-d. Center, visualization of extracted AF via thin-layer chromatography (TLC) from 3-d cultures. Right, relative content of AF quantified from TLC plates.


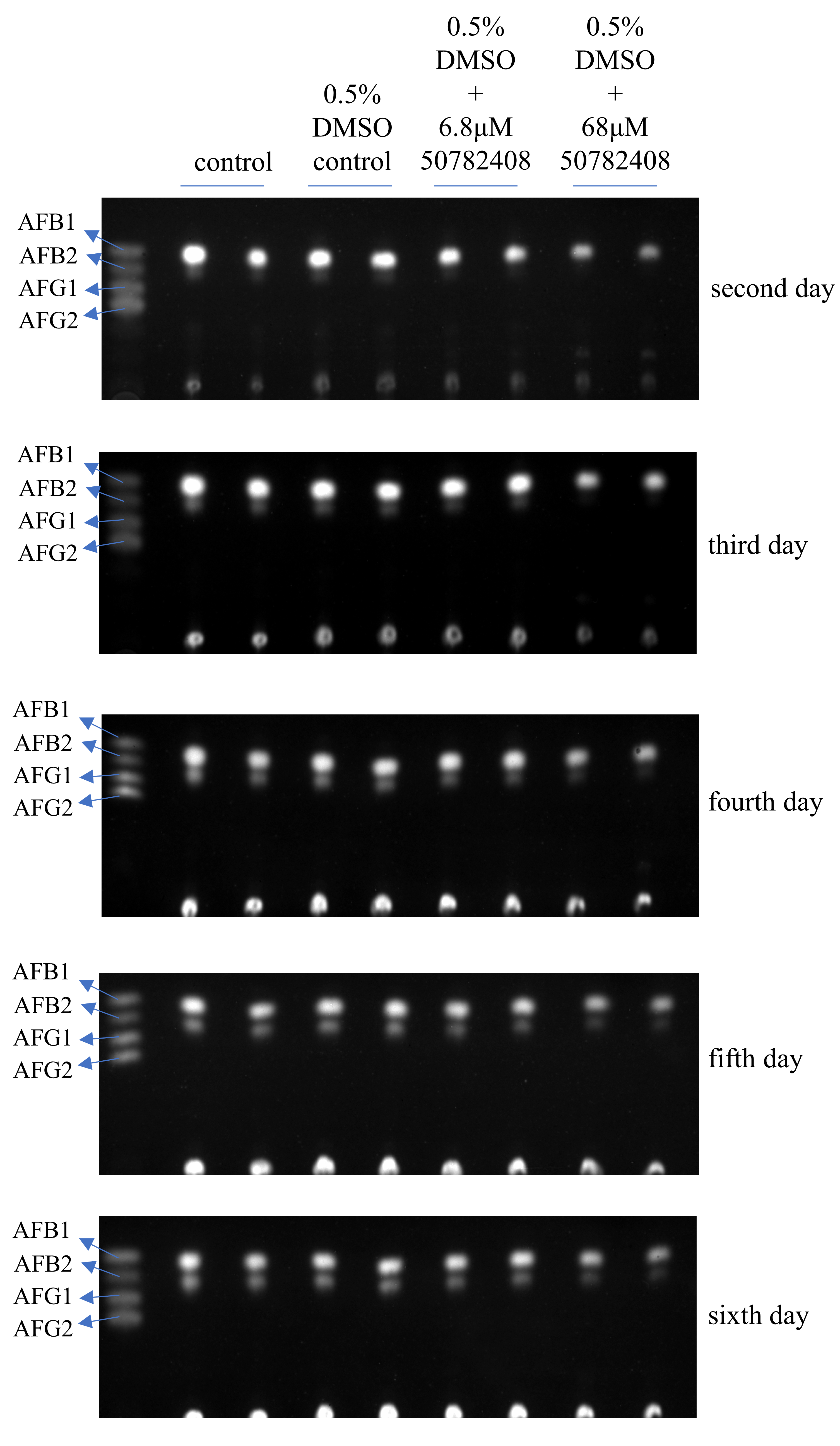


**Figure S5**. TLC of extracted aflatoxins in *A. flavus* culture treated with different concentrations of compound 50782408.


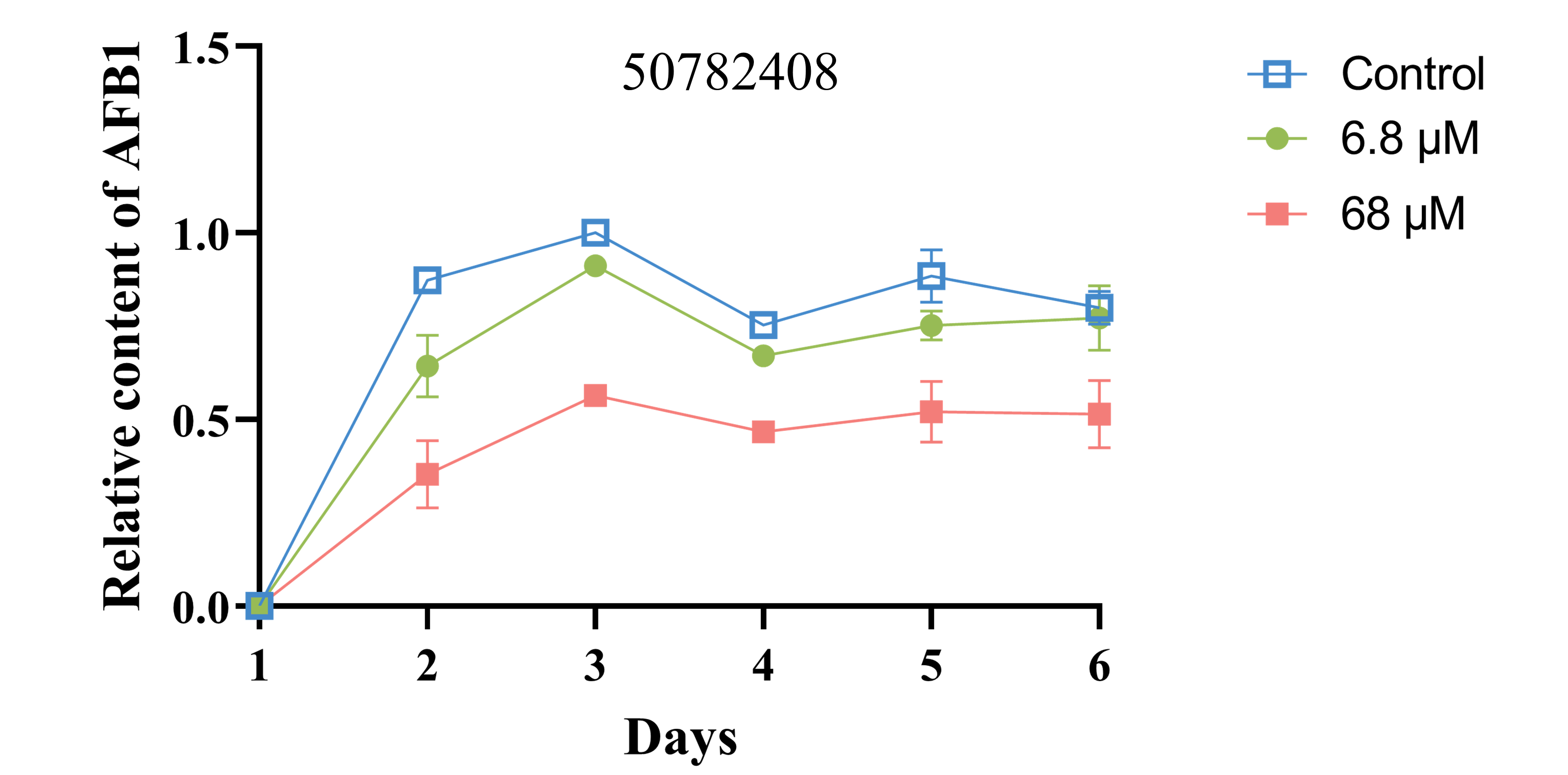


**Figure S6**. Relative content of aflatoxin B1 in *A. flavus* culture treated with different concentrations of compound 50782408.


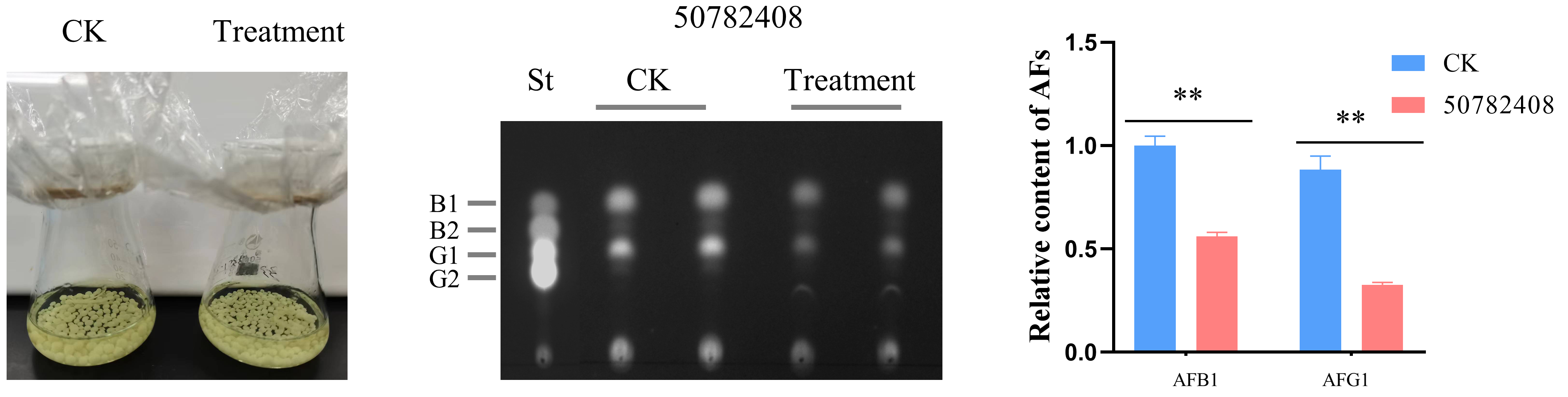


**Figure S7. Effects of compound 50782408 on aflatoxin (AF) biosynthesis in the *Aspergillus parasiticus* NRRL 2999 strain.**


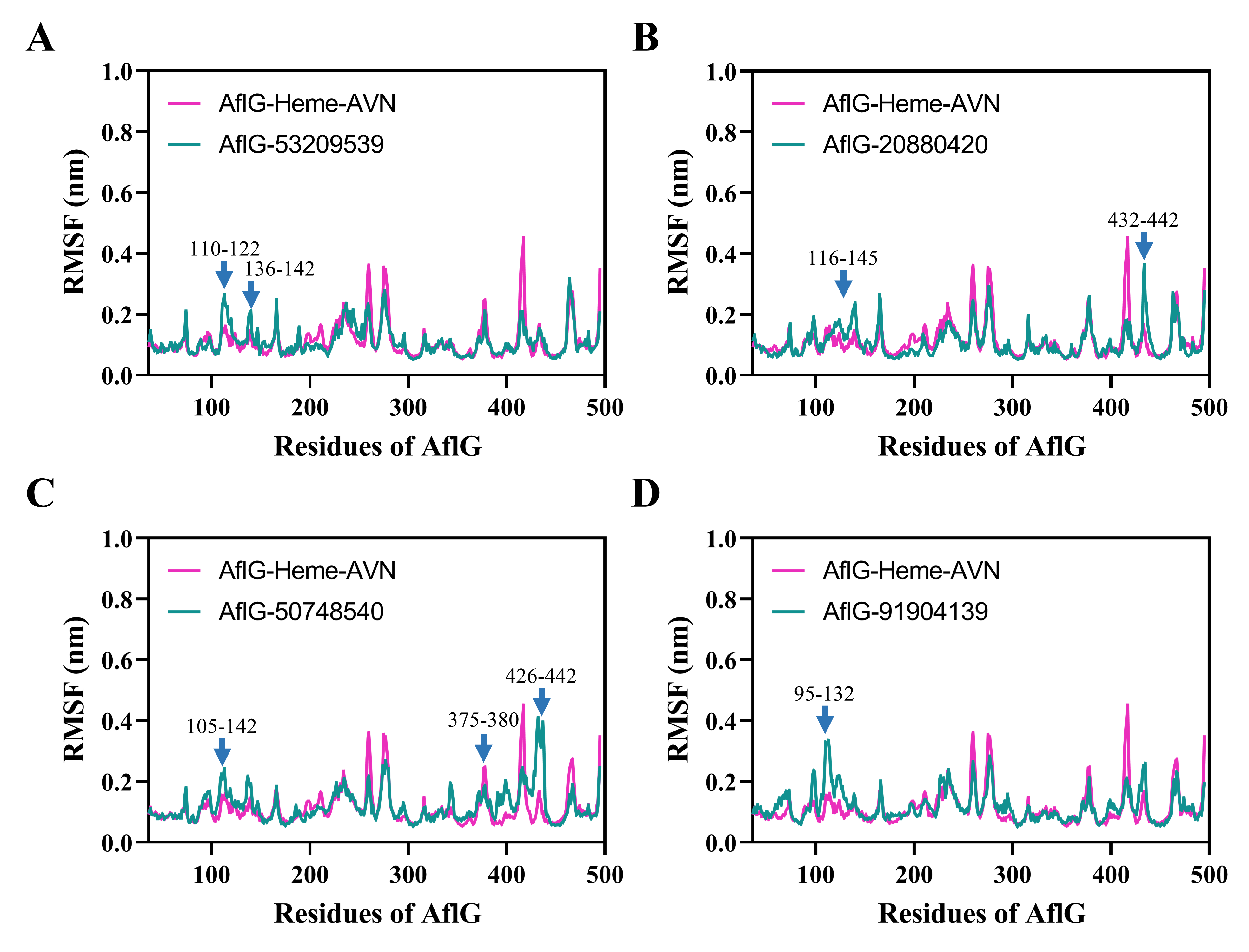


**Figure S8.** **Effects of hit compounds on AflG flexibility.** Root mean square fluctuation (RMSF) plots comparing fluctuations in AflG residues between the AflG-Heme-AVN complex and AflG complex with (A) 53209539, (B) 20880420, (C) 50748540, and (D) 91904139.

**Table S1.** Binding free energies of putative AflG inhibitors from virtual screening. Compounds are identified by their PubChem CID numbers.

| **Compounds** | **Docked pose**  **(kcal/mole)** | **Representative structure from simulations**  **(kcal/mole)** |
| --- | --- | --- |
| 50782408 | -60.25 | -64.93 |
| 53209539 | -58.31 | -62.99 |
| 57336812 | -56.25 | -61.58 |
| 53151533 | -55.46 | -62.11 |
| 20880420 | -55.30 | -61.86 |
| 50748540 | -54.14 | -62.94 |
| 91904139 | -53.27 | -57.06 |
| 54761306 | -53.02 | -55.94 |
